# π-Interrupted Chiral Emitters with Cooperative LE–TADF Emission for Single-Molecule White Circularly Polarized OLEDs

**DOI:** 10.3390/molecules31122195

**Published:** 2026-06-22

**Authors:** Shuang Yang, Wei-Chen Guo, Pei Zhao, Hai-Yan Lu, Chuan-Feng Chen

**Affiliations:** 1 School of Chemical Sciences, University of Chinese Academy of Sciences, Beijing 100049, China; yangshuang21@mails.ucas.ac.cn (S.Y.); guoweichen@iccas.ac.cn (W.-C.G.); 2 Beijing National Laboratory for Molecular Sciences, CAS Key Laboratory of Molecular Recognition and Function, Institute of Chemistry, Chinese Academy of Sciences, Beijing 100190, China

**Keywords:** white circularly polarized electroluminescence, thermally activated delayed fluorescence, single-molecule, dual emission

## Abstract

Single-molecular white circularly polarized luminescence emitters show promise for use in chiral displays and solid-state lighting, but their design remains challenging because broadband emission, exciton utilization, color balance, and chiroptical activity must be integrated within one molecule. Herein, we report a chiral single-molecular white emitter, **DCz-PTZ**, constructed through a π-interrupted strategy by combining a rigid spiro framework, an oxygen-bridged carbazole/cyanobenzene segment, and a phenothiazine donor. The interrupted conjugation suppresses excessive charge-transfer (CT) domination and enables dual emissive channels, including short-wavelength locally excited (LE) emission and long-wavelength CT emission. **DCz-PTZ** exhibits near-ideal white emission in dilute toluene solution with CIE coordinates of (0.33, 0.33), and maintains balanced dual emission in 5 wt% doped films with CIE coordinates of (0.32, 0.34). Photophysical studies support the assignment of the yellow emission to a thermally activated delayed fluorescence (TADF)-active CT state. The enantiomers show mirror-image circularly polarized signals with |*g*_lum_| up to 2.9 × 10^−3^. Optimized white organic light-emitting diodes (WOLEDs) achieve color rendering index (CRI) up to 92 and a maximum external quantum efficiency (EQE_max_) of 1.3%. This work demonstrates a π-interrupted molecular strategy for integrating dual emission, TADF exciton utilization, and circularly polarized electroluminescence (CPEL) in a single chiral emitter.

## 1. Introduction

White organic light-emitting diodes (WOLEDs) have attracted extensive interest for solid-state lighting and display technologies because of their low power consumption, wide viewing angle, mechanical flexibility, and compatibility with large area fabrication [1,2,3,4,5]. Conventional WOLEDs usually rely on multiple emitters to cover the visible region. Although this multicomponent strategy can produce high-quality white emission, it often suffers from complicated device structures, phase separation, uncontrolled energy transfer, and color instability under electrical operation [6,7]. Therefore, single-molecular white emitters, in which multiple emissive channels are integrated into one molecular framework, represent an attractive alternative for simplifying device architecture and improving spectral stability [8,9,10,11,12,13].

Despite this promise, single-molecular white emission remains difficult to realize. White emission requires the coexistence and balance of short-wavelength and long-wavelength emissive components [1,5]. In many systems, the blue component originates from a locally excited (LE) state, whereas the yellow or orange component arises from an intramolecular charge-transfer (ICT) state, an exciplex state, or a triplet-involved delayed emission pathway [14,15]. These emissive channels usually have different radiative rates, exciton utilization efficiencies, and environmental sensitivities. As a result, one channel often dominates the emission, leading to poor color balance, insufficient color rendering, or unstable white output.

Circularly polarized luminescence (CPL) relies on the efficient transfer of molecular chirality to emissive excited states, and CPL materials have shown great promise for applications in three-dimensional displays, information storage, and encryption technologies [16,17,18,19,20,21,22]. The challenge becomes even greater for circularly polarized WOLEDs (CP-WOLEDs) [23,24,25,26,27,28]. At the device level, WOLED operation additionally demands balanced charge transport, efficient exciton recombination, and stable emission under electrical excitation [10,11,15,23,24,29,30,31,32,33,34,35,36,37,38]. Most chiral organic emitters exhibit relatively narrow emission bands or limited dissymmetry factors [26]. Combining single-molecular white emission, thermally activated delayed fluorescence (TADF)-assisted exciton harvesting, and circularly polarized electroluminescence (CPEL) in one molecular system therefore remains a demanding but meaningful target.

Herein, we report a π-interrupted chiral emitter, DCz-PTZ, for single-molecule CP-WOLEDs. DCz-PTZ integrates a rigid spiro framework, an oxygen-bridged carbazole/cyanobenzene segment, and a phenothiazine donor into one molecular scaffold to regulate localized and charge-transfer excited states. This design was inspired by our previous study on the structurally related **SX-PTZ** molecule, which has been accepted for publication (Figure S10). In **SX-PTZ**, the stronger electronic coupling between the emissive segments tends to favor CT-dominated emission and weaken the high-energy LE contribution, making precise color balance difficult. In contrast, the spiro linkage in DCz-PTZ creates a π-interrupted architecture that partially decouples the dicarbazolylcyanobenzene-related segment from the phenothiazine donor. This molecular configuration preserves the LE-like blue-emissive channel while maintaining the lower-energy CT emission, thereby enabling balanced dual emission, TADF-assisted long-wavelength emission, CPL activity, and proof-of-concept CP-WOLED performance within a single chiral emitter.

## 2. Results and Discussion

### 2.1. Molecular Design Strategy

The molecular design of **DCz-PTZ** relies on balancing structural interruption and donor–acceptor interaction. The spiro framework introduces stable chirality and conformational rigidity, while the oxygen-bridged linkage interrupts through bond π-conjugation and suppresses excessive planarization. This interrupted conjugation is expected to prevent complete CT domination, which is a common cause of blue emission loss in single molecular white emitters. Meanwhile, the phenothiazine unit serves as an electron-donating segment, and the cyano-containing aromatic fragment functions as an electron-accepting segment, together forming a moderate CT pathway for long wavelength emission. Therefore, the molecular framework is designed to retain a localized blue-emitting channel while enabling a lower-energy phenothiazine involved CT channel.

### 2.2. Synthesis and Thermal Properties

**Scheme 1:**
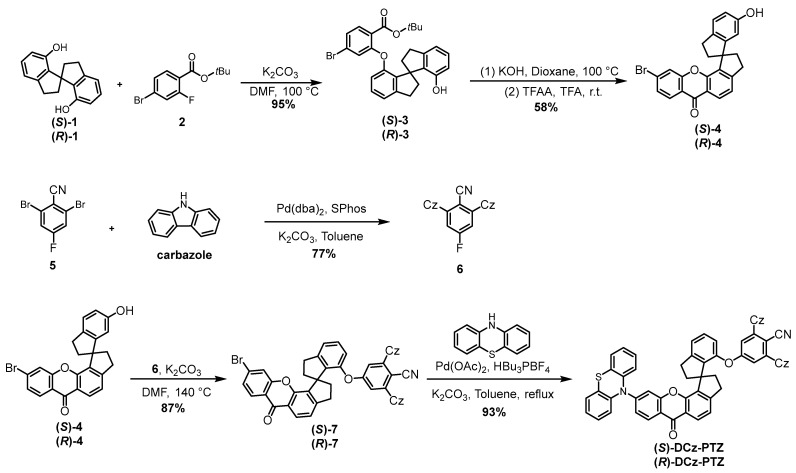
Synthesis routes of (*S*/*R*)-**DCz-PTZ**.

To experimentally validate this molecular design, both enantiomers of **DCz-PTZ** were prepared and fully characterized, followed by evaluation of their thermal stability for vacuum-deposited OLED fabrication. The enantiomeric emitters, (*R*)-**DCz-PTZ** and (*S*)-**DCz-PTZ**, were synthesized through a multistep route involving nucleophilic substitution, Friedel-Crafts acylation, and Buchwald-Hartwig coupling. The commercially available chiral scaffold was used as the key starting structure, allowing the chiral configuration to be introduced at an early stage. The synthetic sequence proceeded with good yields and reproducibility, demonstrating the practical accessibility of this molecular platform (Scheme 1).

Thermogravimetric analysis shows that **DCz-PTZ** possesses excellent thermal stability, with a 5% weight-loss decomposition temperature of 448 °C under nitrogen (Figure S1). Such high thermal robustness is beneficial for vacuum thermal evaporation and stable OLED fabrication.

### 2.3. Photophysical Properties

The UV-vis absorption spectrum of **DCz-PTZ** in dilute toluene solution exhibits multiple absorption bands (Figure S2). The absorption region around 300–350 nm is mainly assigned to π–π* transitions of the carbazole and phenothiazine units. The weak lower-energy absorption band can be attributed to CT transitions, indicating donor–acceptor electronic coupling in the ground state.

**Figure 1:**
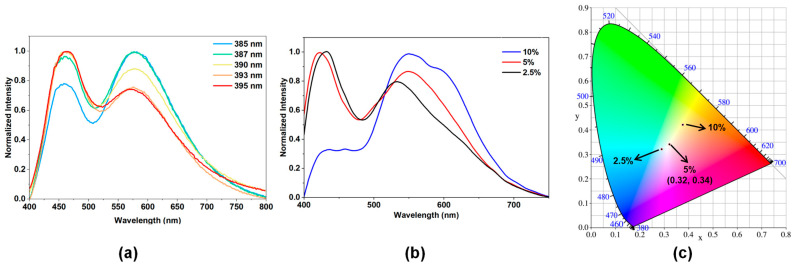
(**a**) Spectra of **DCz-PTZ** in dilute toluene solution at different excitation wavelengths at 298 K. (**b**) Normalized fluorescence spectrum of **DCz-PTZ** films with different doping ratios at 298 K. (**c**) CIE coordinate plots of **DCz-PTZ** films with different doping ratios.

The photoluminescence spectra show characteristic dual-band emission. In dilute toluene solution, **DCz-PTZ** exhibits two emission bands at approximately 458 and 580 nm. The short-wavelength band is relatively narrow, while the long-wavelength band is broader and red-shifted, suggesting different excited-state characters. Under 390 nm excitation, these two bands reach a balanced intensity ratio, producing near-white photoluminescence with CIE coordinates of (0.33, 0.33) (Figure 1a). Excitation spectra monitored at 458 and 580 nm further show distinct excitation responses (Figure S3), indicating that the two emission bands originate from different emissive channels within the same molecule. The high-energy emission band at 458 nm can be assigned to an LE-like emission associated with the dicarbazolylcyanobenzene-related segment, while the long-wavelength emission is mainly attributed to a CT emissive channel. This assignment is further supported by the theoretical excited-state characterization discussed below.

The solid-state emission behavior was then investigated by doping **DCz-PTZ** into a 2,8-bis(diphenylphosphoryl)dibenzo[b,d]thiophene (PPT) host. The doped films retain dual-band emission with peaks around 420 and 550 nm (Figure 1b), confirming that the dual-emission character is preserved in the host matrix. The relative intensity of the two bands varies with doping concentration. At lower doping concentrations, the short-wavelength component is more pronounced, whereas increasing the doping concentration enhances the long-wavelength component, likely due to stronger intermolecular interactions and stabilization of the CT-like excited state. At 5 wt% doping, the two emission components show the most suitable balance, giving white emission with CIE coordinates of (0.32, 0.34) (Figure 1c). The retention of the short-wavelength component in doped films suggests that the π-interrupted and sterically hindered framework effectively suppresses excessive aggregation-induced CT domination in the solid state.

**Figure 2:**
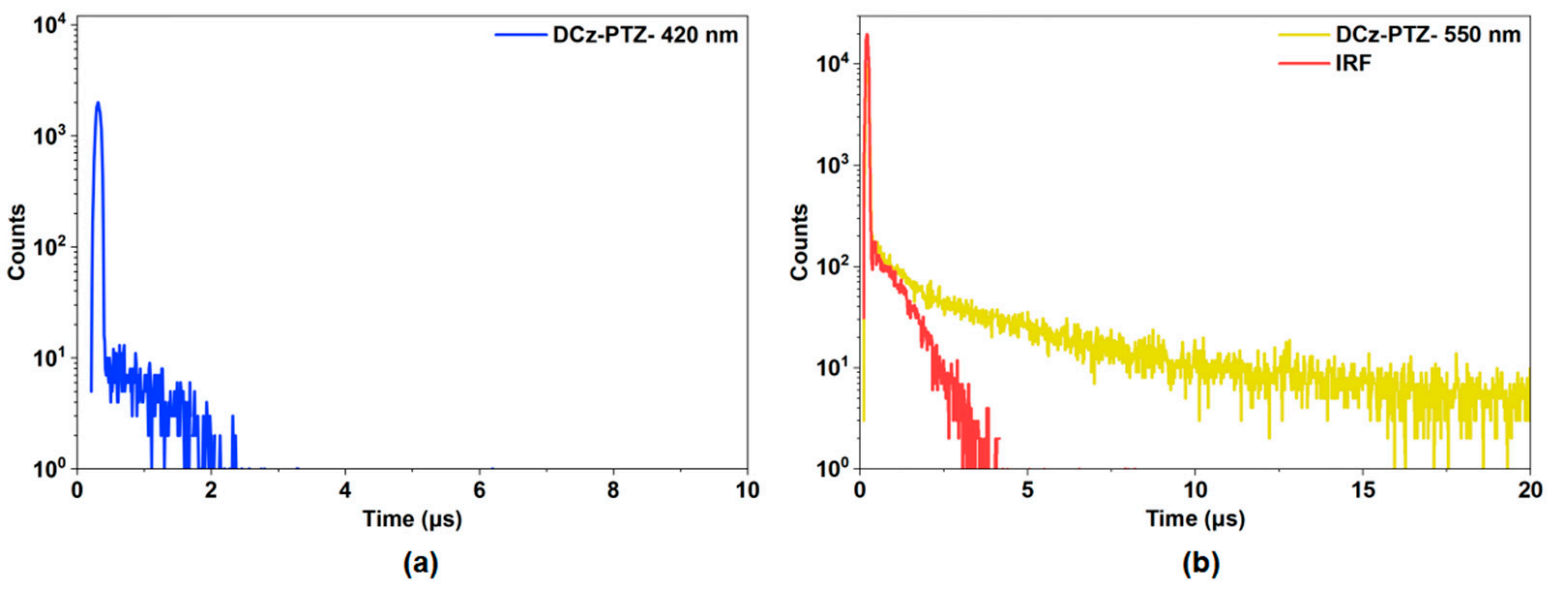
Transient PL decay curves of **DCz-PTZ** in 5 wt% doped films at (**a**) 420 nm and (**b**) 550 nm at 298 K.

Temperature-dependent steady-state photoluminescence (PL) spectra provide further insight into the different emissive channels (Figure S4). Upon cooling from 298 to 78 K, the blue emission around 420 nm remains relatively stable, whereas the yellow emission around 550 nm is significantly weakened, indicating that the long-wavelength component is thermally activated. Transient PL decay measurements monitored at 420 and 550 nm further support this assignment (Figure 2). The 420 nm decay closely follows the IRF and rapidly decays within the nanosecond regime, indicating that this emission is dominated by prompt fluorescence (Figure S5), whereas that in the yellow-emission region exhibits both short-lived and delayed components, with lifetimes of approximately 0.72 and 4.9 μs, respectively. The delayed component increases with increasing temperature, confirming the involvement of thermally activated reverse intersystem crossing (RISC) in the long-wavelength emission channel (Figure S6).

Low-temperature fluorescence and phosphorescence spectra measured at 77 K afford an experimental Δ*E*_ST_ of 0.12 eV for the yellow-emitting CT channel (Figure S7). Although this value is larger than that of many highly efficient TADF emitters, it remains sufficiently small to support thermally assisted triplet upconversion. Together with the spectral profiles, temperature-dependent PL, and transient decay results, these data support a cooperative dual-emission mechanism in which a stable blue LE fluorescence channel and a yellow TADF-active CT channel operate within the same π-interrupted molecular framework. The photoluminescence quantum yield (PLQY) increases from 36% in the neat film to 51% in the 5 wt% doped film (Table 1), indicating that the host matrix suppresses concentration-induced nonradiative decay while maintaining the dual-emission character.

### 2.4. Theoretical Calculations

To further rationalize the experimentally observed dual-emission behavior and clarify the roles of the dicarbazolylcyanobenzene segment and the π-interrupted spiro framework, density functional theory (DFT) and time-dependent DFT (TD-DFT) calculations were performed for DCz-PTZ (Figure S9). The calculated HOMO is mainly localized on the phenothiazine donor unit, whereas the LUMO is primarily distributed over the acceptor segment, indicating a typical donor-acceptor electronic structure. The calculated HOMO and LUMO energy levels are −5.28 and −1.88 eV, respectively, corresponding to an energy gap of approximately 3.40 eV. Such a spatially separated frontier orbital distribution provides the electronic basis for the CT character of the low-energy emissive channel and is also beneficial for reducing the singlet-triplet exchange energy of the CT state. The optical band gap estimated from the absorption onset is approximately 3.18 eV, which is smaller than the calculated HOMO–LUMO gap of 3.40 eV. This difference is reasonable because the DFT-derived HOMO–LUMO gap represents a ground-state orbital-energy difference, whereas the optical gap reflects the lowest optically accessible excited state.

NTO analysis was then performed to further distinguish the excited state characters involved in the dual emission. (Figure S11) For the lower-energy excited state, the hole is mainly located on the phenothiazine donor, while the electron is distributed over the acceptor region, suggesting effective intramolecular charge transfer from the donor to the acceptor segment upon excitation. This result supports the assignment of the long-wavelength yellow emission to a CT emissive channel, which is consistent with its TADF behavior observed experimentally. In contrast, the high-energy excited state associated with the blue emission shows largely overlapped hole and particle distributions mainly localized on the dicarbazolylcyanobenzene-related framework. This localized distribution is clearly different from a typical long-range CT transition, in which the hole and particle are spatially separated over donor and acceptor fragments. Therefore, the 458 nm emission is more appropriately assigned to an LE-like emissive channel originating from the dicarbazolylcyanobenzene-related segment.

To further clarify the function of the π-interrupted spiro framework, the present molecule was compared with the structurally related **SX-PTZ** molecule from our previous work, which has been accepted for publication (Figure S10 and Table S1). In **SX-PTZ**, the π-interrupted configuration is absent or much less effective, leading to stronger electronic coupling between the donor and acceptor/emissive segments. The calculated HOMO is distributed over the PTZ donor and the adjacent aromatic framework, whereas the LUMO is mainly located on the acceptor segment. The corresponding NTO analysis shows pronounced spatial separation between the hole and electron distributions, indicating a more CT-dominated excited-state character. This computational result agrees with the PL behavior of **SX-PTZ** observed in our previous study, where the blue-emission component was obviously insufficient.

By comparison, the spiro linkage in DCz-PTZ partially decouples the dicarbazolylcyanobenzene-related segment from the phenothiazine donor, thereby suppressing excessive CT domination and preserving the high-energy LE-like blue-emissive channel while maintaining the lower-energy CT channel. These theoretical results demonstrate that the dicarbazolylcyanobenzene segment is not merely a structural unit, but an electronically active high-energy emissive segment. Together with the temperature-dependent PL, transient decay, and low-temperature fluorescence/delayed-emission measurements discussed above, the calculations confirm that the balanced dual emission of DCz-PTZ originates from the coexistence of a DCz-CN-related LE-like channel and a PTZ-related CT channel, enabling single-molecule white-light emission.

### 2.5. Chiroptical Properties

After establishing the LE/CT dual-emission mechanism, circular dichroism (CD) and CPL spectroscopy were used to examine whether the chirality could be effectively transferred to the emissive excited states. Owing to the rigid spiro framework, (*R*)-**DCz-PTZ** and (*S*)-**DCz-PTZ** possess stable chiral configurations. The CD spectra of the dilute toluene solution exhibit clear mirror-image Cotton effects (Figure S8), confirming the successful construction of enantiomeric chiral emitters. Strong CD signals appear in the 280–350 nm region, corresponding mainly to aromatic π-π* transitions.

**Figure 3:**
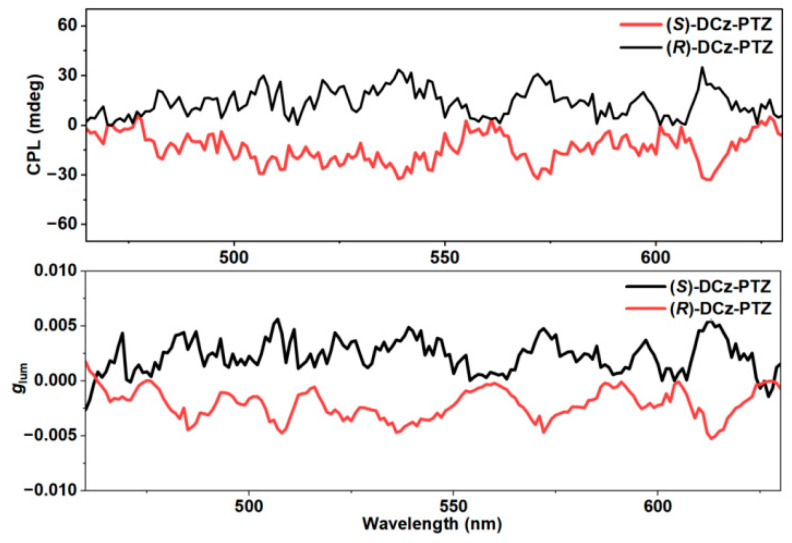
CPL spectra and wavelength-dependent glum curves of (*R*/*S*)-**DCz-PTZ** in doped films.

The mirror-image CPL spectra demonstrate effective chirality transfer from the framework to the emissive excited states (Figure 3). The maximum |*g*_lum_| reaches 2.9 × 10^−3^ in doped films, indicating a distinct CPL response from the dual-emission system.

### 2.6. Electroluminescence Property

**Figure 4:**
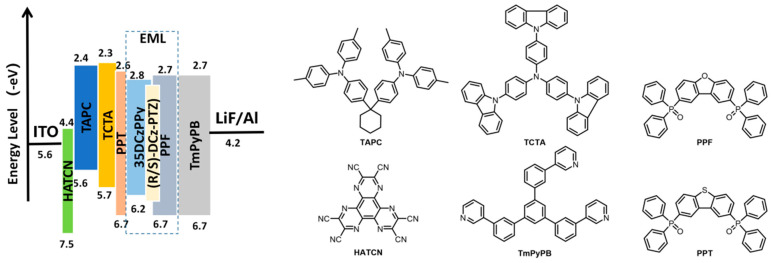
Energy-level diagram of device W4 and chemical structure of each layer material.

**Figure 5:**
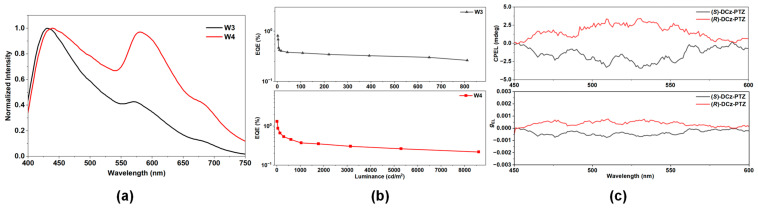
(**a**) The electroluminescence (EL) spectrum of devices W3 and W4 at 8.5 V. (**b**) EQE luminance characteristic curves of devices W3 and W4. (**c**) CPEL spectrum and *g*_EL_ wavelength-dependent curves of (*R*/*S*)-**DCz-PTZ**.

Encouraged by the balanced white photoluminescence and clear CPL activity in doped films, vacuum-deposited WOLEDs were fabricated to evaluate the electroluminescence and CPEL performance of **DCz-PTZ**. Device W1 was fabricated with the following structure: ITO/(1,1-Bis[(di-4-tolylamino)phenyl]cyclohexane) TAPC/tris(4-carbazoyl-9-ylphenyl)amine (TCTA)/PPT:Emitter/(1,3,5-Tris(3-pyridyl-3-phenyl)benzene) TmPyPB/LiF/Al. This device shows a high turn-on voltage of 12 V and low EQEmax. These results indicate inefficient charge injection and unbalanced carrier recombination. To improve hole injection, HATCN was introduced in W2: ITO/HATCN/TAPC/TCTA/PPT:Emitter/TmPyPB/LiF/Al. Compared with W1, W2 exhibits a reduced turn-on voltage of 5.2 V and an increased EQE_max_ of 0.4%, confirming improved hole injection and carrier balance. Although W2 showed improved charge injection, further optimization of the spectral balance was required to improve the CRI. Therefore, an ultrathin PPT interlayer was introduced in W3: ITO/HATCN/TAPC/TCTA/PPT/PPT:5 wt% Emitter/TmPyPB/LiF/Al. W3 shows a lower turn-on voltage and dual emission around 470 and 576 nm. The device achieves a high CRI of 92, CIE coordinates of (0.30, 0.29), *L*_max_ = 810 cd m^−2^, and CCT = 7408 K. The high CRI is attributed to improved spectral balance between blue and yellow emission channels, enabled by interfacial regulation of carrier recombination and exciton distribution. To further enhance brightness and efficiency, a dual-emitting-layer architecture was used in W4: ITO/HATCN/TAPC/TCTA/PPT/3,5-bis(3-(9H-carbazol-9-yl)phenyl)pyridine (35DCZPPy):5 wt% Emitter/(2,8-Bis(diphenyl-phosphoryl)-dibenzo[b,d]furan) PPF:5 wt% Emitter/TmPyPB/LiF/Al (Figure 4). W4 exhibits the best overall device performance, with a turn-on voltage of 3.7 V, EQE_max_ of 1.3%, and *L*_max_ of 8600 cd m^−2^. The device shows dual emission around 440 and 580 nm, CIE coordinates of (0.33, 0.32), CRI of 88, and CCT of 10,487 K (Figure 5). Compared with W3, W4 exhibits significantly enhanced brightness and EQE owing to improved host-assisted charge balance and exciton confinement. However, the stronger blue component in W4 leads to a higher CCT and slightly lower CRI. Therefore, W3 represents the color-quality-optimized device, whereas W4 represents the efficiency -brightness-optimized device. Although the EQE_max_ of 1.3% is still lower than those of highly optimized monochromatic TADF OLEDs, the present devices should be evaluated in the context of single-molecule CP-WOLEDs, where white-emission balance, color quality, chiroptical activity, and electroluminescence efficiency must be simultaneously achieved in one emitter. Device W3 affords high-quality white emission with a CRI of 92, whereas device W4 gives improved brightness and efficiency with an EQE_max_ of 1.3% while retaining CPEL activity. These results indicate that the π-interrupted chiral molecular design provides a feasible strategy for constructing single-molecule CP-WOLEDs. Further improvements in device efficiency are expected through optimization of host materials, carrier balance, exciton confinement, and optical outcoupling. The current density–voltage–luminance (J–V–L) characteristics of W3 and W4 are shown in Figure S12.

Based on the optimized W4 architecture, CPEL was further measured using W4-*R* and W4-*S* devices fabricated from the corresponding enantiomers. The two devices exhibit clear mirror-image CPEL signals, indicating that the chirality of DCz-PTZ is preserved under electrical excitation. The maximum |*g*_EL_| reaches 9.0 × 10^−4^_,_ confirming that the molecular chirality of **DCz-PTZ** can be retained under electrical excitation (Figure 5 and Table 2).

## 3. Materials and Methods

The starting materials and other reagents were procured from commercial sources and used without any additional purification. ^1^H spectra were recorded on AVIII 300, 400 and 500 MHz spectrometers in CDCl_3_ solution. High-resolution mass spectra were measured on a Thermo Fisher^®^ (Waltham, MA, USA) Exactive high-resolution LC-MS spectrometer. The thermogravimetric analysis (TGA) was performed on a Q600 SDT (New Castle, DE, USA) thermal analyzer with a heating rate of 10 °C/min in nitrogen atmosphere.

UV-vis spectra were acquired using a PerkinElmer^®^ (Waltham, MA, USA) UV/Vis/NIR spectrometer (Lambda 950). The photoluminescence spectra and transient PL decay characteristics were measured utilizing an Edinburgh Instruments (Livingston, UK) FLS 1000 spectrometer. The absolute PLQY was determined on the FLS (Livingston, UK) 1000 spectrometer employing an integrating sphere with excitation at a wavelength of 360 nm. The CD spectra were recorded on a JASCO (Tokyo, Japan) J810 spectropolarimeter. The CPL and CPEL measurements were conducted utilizing a commercialized instrument JASCO (Tokyo, Japan) CPL-300 spectrophotometer at room temperature. Theoretical calculations were carried out with the Gaussian 09 software package [38]. Excited states using density functional theory (DFT) and time-dependent DFT (TD-DFT) at the B3LYP-D3(BJ) level [39,40,41,42,43]. The molecule was optimized at the B3LYP-D3/6-31G(d) with DFT using Gaussian program. Unless otherwise specified, the calculations were conducted in the gas phase.

The electroluminescence properties were measured using OLED devices. The anode substrate utilized was a glass coated with Indium tin oxide (ITO), possessing a sheet resistance of 10 Ω per square. Before device fabrication, the ITO glass substrates underwent a thorough cleaning process involving Decon 90 treatment, followed by rinsing in ultrapure water and ethanol. Subsequently, they were dried at 120 °C in an oven and subjected to two minutes O_2_ plasma treatment to enhance the surface work function of the ITO anode. Subsequently, all organic functional layer materials, Lithium Fluoride (LiF), and an Aluminum (Al) were evaporated consecutively onto the ITO glass substrates in a vacuum deposition chamber. The electroluminescence and current-voltage luminance characteristics of the devices were measured using a computer-controlled Spectrascan (North Syracuse, NY, USA) PR 670 spectrophotometer and Keithley (Cleveland, OH, USA) 2400 SourceMeter subsequent to device packaging.

All newly synthesized intermediates and target enantiomers were characterized by ^1^H NMR, ^13^C NMR, and high-resolution mass spectrometry (HRMS). Detailed synthetic procedures, spectral assignments, and copies of NMR spectra are provided in the Supplementary Materials.

## 4. Conclusions

In summary, **DCz-PTZ** was developed as a π-interrupted chiral emitter that integrates blue LE fluorescence, yellow TADF-active CT emission, and circularly polarized emission within one molecular framework. The emitter shows near-white PL in both solution and 5 wt% doped films, with CIE coordinates of (0.33, 0.33) and (0.32, 0.34), respectively. Photophysical measurements and theoretical analysis support the cooperative LE/CT emission mechanism, while the enantiomers exhibit mirror-image CPL signals with |*g*_lum_| up to 2.9 × 10^−3^. In WOLEDs, W3 achieves a CRI of 92, whereas W4 reaches EQE_max_ = 1.3%, CIE = (0.33, 0.32), and |*g*_EL_| = 9.0 × 10^−4^. These results demonstrate the feasibility of using π-interrupted chiral emitters for single-molecular CP-WOLEDs.

## Figures and Tables

**Table 1:** Summary of the photophysical properties of **DCz-PTZ**.

Compound	λ_PL_^1^ (nm)	Φ_PL_^2,3^ (%)	Δ*E*_ST_^4^ (eV)	τ_short_^5^ (μs)	Τ_d_^6^ (μs)
**DCz-PTZ**	420, 550	36, 51	0.12	0.720 ± 0.045	4.92 ± 0.21

^1^ The maximum emission wavelength of **DCz-PTZ** measured in a 5 wt% doped film. ^2,3^ The PLQY of **DCz-PTZ** measured in the neat film and doped film, respectively. ^4^ The Δ*E*_ST_ of the yellow emission band of **DCz-PTZ** measured in doped film. Δ*E*_ST_ was estimated from the emission maxima. The delay time used for phosphorescence acquisition was 100 μs. ^5^ The prompt lifetimes of the yellow emission bands, respectively. ^6^ The delayed lifetime of the yellow emission band.

**Table 2:** Summary of Device Performance of CP-WOLEDs for **DCz-PTZ**.

Device	W1	W2	W3	W4
*V*_on_ ^1^ (V)	12	5.2	4.0	3.7
EQE_max_ ^2^ (%)	0.02	0.4	0.3	1.3
CE_max_ ^3^ (cd/A)	0.014	1.38	1.27	1.58
PE_max_ ^4^ (lm/W)	0.054	1.71	1.42	4.28
*L*_max_ ^5^ (cd/m^2^)	503	845	810	8600
|*g*_EL_| ^6^ (×10^−3^)	-	-	-	0.9
CIE ^7^	(0.33, 0.41)	(0.29, 0.33)	(0.30, 0.29)	(0.33, 0.32)
CRI ^8^	73	84	92	88
CCT ^9^ (K)	4707	8408	7408	10,487

^1^ *V*_on_: operating voltage at a brightness of 1 cd/m^2^. ^2^ Maximum external quantum efficiency. ^3^ Maximum current efficiency. ^4^ Maximum power efficiency. ^5^ Maximum brightness. ^6^ Electroluminescence asymmetry factor. ^7^ International Commission on Illumination 1931 coordinates. ^8^ Color rendering index. ^9^ Correlated color temperature.

## Data Availability

Data are included in the article/Supplementary Materials/referenced in the article.

## Supplementary Materials

The following supporting information can be downloaded at: https://www.mdpi.com/article/10.3390/molecules31122195/s1, Figures S1–S20, Scheme S1 and Tables S1 and S2 are cited in Supplementary Materials. Scheme S1. Synthesis of (*S*)-**DCz-PTZ**/(*R*)-**DCz-PTZ**. Figure S1. The thermogravimetric analysis of **DCz-PTZ**. Figure S2. UV-vis absorption spectrum of **DCz-PTZ** in toluene solution (c = 1.0 × 10^−5^ M, L = 1 cm). The maximum molar absorption coefficient was estimated to be 5.91 × 10^4^ M^−1^cm^−1^ according to the Beer-Lambert law. Figure S3. Excitation spectra of **DCz-PTZ** at different emission wavelengths in dilute toluene solution at 298 K. Figure S4. Temperature dependent steady-state fluorescence spectra of **DCz-PTZ** in doped films. Figure S5. High-resolution transient PL decay profile of **DCz-PTZ** monitored at 420 nm with the corresponding instrument response function (IRF). Figure S6. Temperature dependent transient fluorescence spectra of **DCz-PTZ** in doped films. Figure S7. Fluorescence and phosphorescence spectra of **DCz-PTZ** in doped films at 77 K. Figure S8. CD spectrum of (*R*/*S*)-**DCz-PTZ** in dilute toluene solution at 298 K. Figure S9. Corresponding HOMO, LUMO, electron and hole distributions and calculated energy levels of **DCz-PTZ** based on optimized structure (DFT with B3LYP functional and the 6-31G(d) basis set). Figure S10. Calculated HOMO/LUMO distributions and NTO hole/electron distributions of the reference molecule **SX-PTZ** without effective π-interrupted/spiro decoupling. The spatial separation between the hole and electron distributions indicates a pronounced CT character. Combined with the insufficient blue-emission component observed in its PL spectrum, this result suggests that the absence of effective π interruption leads to CT-dominated emission and suppresses the high-energy locally excited component. Figure S11. Natural transition orbital distributions of the T1 and T3 excited states of **DCz-PTZ** calculated by TD-DFT. Figure S12. (a) Current density-voltage-Luminance (J-V-L) characteristics of devices W3 (b) *J-V-L* characteristics of devices W4. Figure S13. ^1^H NMR spectrum of (*S*)-3/(*R*)-3 (500 MHz, CDCl_3_). Figure S14. ^13^C NMR spectrum of (*S*)-3/(*R*)-3 (126 MHz, CDCl_3_). Figure S15. ^1^H NMR spectrum of (*S*)-4/(*R*)-4 (300 MHz, CDCl_3_). Figure S16. ^13^C NMR spectrum of (*S*)-4/(*R*)-4 (100 MHz, CDCl_3_). Figure S17. 1H NMR spectrum of compound 6 (300 MHz, CDCl_3_). Figure S18. ^13^C NMR spectrum of compound 6 (100 MHz, CDCl_3_). Figure S19. ^1^H NMR spectrum of compound (*S*)-**DCz-PTZ**/*(R*)-**DCz-PTZ** (500 MHz, CDCl_3_). Figure S20. ^13^C NMR spectrum of compound (*S*)-**DCz-PTZ**/(*R*)-**DCz-PTZ** (126 MHz, CDCl_3_). Table S1. Chiroptical properties of representative chiral TADF emitters. Table S2. Comparison of excited-state characteristics and PL behavior between **DCz-PTZ** and the reference molecule **SX-PTZ**. References [39,40,41,42,43,44] are cited in the Supplementary Material.
