# Supplementary material for: π-Interrupted Chiral Emitters with Cooperative LE–TADF Emission for Single-Molecule White Circularly Polarized OLEDs
Source: Molecules. 2026 Jun 22;31(12):2195. doi: 10.3390/molecules31122195 (PMC13305632; doi:10.3390/molecules31122195)
Supplement: Supplementary file 1 [file molecules-31-02195-s001.zip › molecules-4381770-supplementary.pdf]

Supplementary Materials

π-Interrupted Chiral Emitters with Cooperative LE–TADF Emission for Single-Molecule White Circularly Polarized OLEDs

Shuang Yang, Wei-Chen Guo, Pei Zhao, Hai-Yan Lu,* and Chuan-Feng Chen**

1. General Information

The starting materials and other reagents were procured from commercial sources and used without any additional purification. ^{1}H spectra were recorded on AVIII 300, 400 and 500 MHz spectrometers in $CDCl_3$ solution. High-resolution mass spectra were measured on a Thermo Fisher® Exactive high resolution LC-MS spectrometer. The thermogravimetric analysis (TGA) was performed on a Q600 SDT thermal analyzer with a heating rate of 10 ºC/min in nitrogen atmosphere.

UV-vis spectra were acquired using a PerkinElmer® UV/Vis/NIR spectrometer (Lambda 950). The photoluminescence spectra and transient PL decay characteristics were measured utilizing an Edinburgh Instruments FLS 1000 spectrometer. The absolute photoluminescence quantum yield (PLQY) was determined on the FLS 1000 spectrometer employing an integrating sphere with excitation at a wavelength of 360 nm. The circular dichroism (CD) spectra were recorded on a JASCO J810 spectropolarimeter. The circularly polarized photoluminescence (CPL) and circularly polarized electroluminescence (CPEL) measurements were conducted utilizing a commercialized instrument JASCO CPL-300 spectrophotometer at room temperature. Theory calculation was carried out with the Gaussian 09 software package.[S1] Excited states using density functional theory (DFT) and timedependent DFT (TD-DFT) at the B3LYP-D3(BJ) level.[S2-S5] The molecule was optimized at the B3LYP-D3/6-31G(d) with DFT using Gaussian program.

The electroluminescence properties were measured using OLEDs devices. The anode substrate utilized was a glass coated with Indium tin oxide (ITO), possessing a sheet resistance of 10 Ω per square. Before device fabrication, the ITO glass substrates underwent a thorough cleaning process involving Decon 90 treatment, followed by rinsing in ultrapure water and ethanol. Subsequently, they were dried at 120 °C in an oven and subjected to two minutes O_2 plasma treatment to enhance the surface work function of the ITO anode. Subsequently, all organic functional layer materials, Lithium Fluoride (LiF), and an Aluminum (Al) were evaporated

consecutively onto the ITO glass substrates in a vacuum deposition chamber. The electroluminescence and current-voltage luminance characteristics of the devices were measured using a computer-controlled Spectrascan PR 670 spectrophotometer and Keithley 2400 SourceMeter subsequent to device packaging.

2. Experimental Procedures

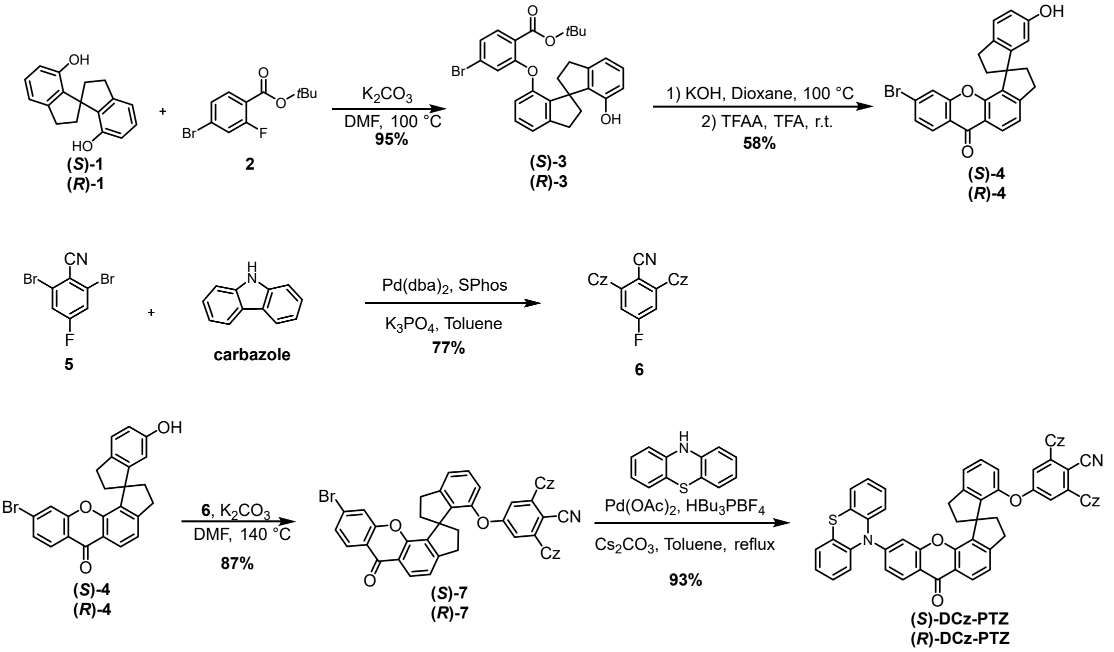

Scheme S1. Synthesis of **(*S*)-DCz-PTZ/(*R*)-DCz-PTZ**.

Synthesis of **(*S*)-3/(*R*)-3**.

(*S*)/(*R*)-1 (2.52 g, 10 mmol) and potassium carbonate (K_2CO_3, 4.15 g, 30 mmol) were dissolved in 30 mL of anhydrous dimethylformamide (DMF), then tert-butyl 4-bromo-2-fluorobenzoate (2.2 g, 10mmol) were added to the reaction and the mixture were heated to 100 °C for 12 hour. After cooling to room temperature, 80 mL of saturated NH_4Cl aqueous solution were added to the mixture, extracted with ethyl acetate for three times. The combined organic phases were dried over anhydrous Na_2SO_4 and concentrated in vacuo. The resultant crude product was purified by column chromatography (PE/EA = 5/1, v/v) to afford the desired product as white solid.

For **(*S*)-3** (4.81 g, 95%)/**(*R*)-3** (4.76 g, 94%): **^{1}H NMR** (500 MHz, $CDCl_3$) δ 7.51 (d, *J* = 8.4 Hz, 1H), 7.18 (t, *J* = 7.8 Hz, 1H), 7.12 (dd, *J* = 8.4, 1.8 Hz, 1H), 7.07 (d, *J* = 7.4 Hz, 1H), 6.90 (t, *J* = 7.7 Hz, 1H), 6.85 (s, 1H), 6.68 (d, *J* = 7.4 Hz, 1H), 6.54 (d, *J* = 8.0 Hz, 1H), 6.43 (d, *J* = 7.9 Hz, 1H), 3.15 – 3.09 (m, 2H), 3.03 – 2.97 (m, 2H), 2.63 – 2.54 (m, 1H), 2.46 – 2.37 (m, 1H), 2.35 – 2.22 (m, 2H), 1.44 (s, 9H). **^{13}C NMR** (126 MHz, $CDCl_3$) δ 164.2, 156.4, 153.6, 152.1, 147.0, 145.2, 132.3, 129.4, 128.4, 126.5, 126.1, 123.6, 123.1, 120.6, 117.3, 117.0, 114.0, 77.4, 77.2, 76.9, 58.9, 39.0, 38.6, 31.6, 28.2. **HRMS** (APCI) **M+H$^+$** Calcd for $C_{28}H_{28}BrO_4^+$: 508.1019 Found: 508.1014.

Synthesis of **(*S*)-4**/**(*R*)-4**.

(*S*)-3/**(*R*)-3** (4.6 g, 9 mmol) and potassium hydroxide (5.05 g, 90 mmol) were dissolved in the mixture of dioxane (80 mL) and distilled water (40 mL). The reaction was heated to 100 °C for 12 hours. After the reaction cooling to room temperature, hydrochloric acid (1.0 M) was used to neutralize the solution. The solid was filtered and washed with water and petroleum ether (PE) to afford white solid without other more purification. White solid was dissolved in 40 mL trifluoroacetic acid, and trifluoroacetic anhydride (13.2 g, 63 mmol) were added dropwise to the reaction at 0 °C. After stirring at room temperature overnight, the mixture was slowly added to saturated NaHCO3 aqueous solution. Then extraction with DCM and purification by column chromatography (PE/EA = 4/1, v/v), **(*S*)-4**/**(*R*)-4** can be afforded as white solid.

For **(*S*)-4** (2.26 g, 58%)/**(*R*)-4** (2.25 g, 58%): **^{1}H NMR** (300 MHz, $CDCl_3$) δ 8.2 (d, *J* = 8.1 Hz, 1H), 8.1 (d, *J* = 8.5 Hz, 1H), 7.4 (dd, *J* = 8.5, 1.7 Hz, 1H), 7.4 – 7.4 (m, 2H), 7.3 (d, *J* = 8.0 Hz, 1H), 7.0 – 6.9 (m, 1H), 6.9 (d, *J* = 1.8 Hz, 1H), 3.2 (ddd, *J* = 12.7, 9.6, 4.4 Hz, 4H), 2.5 – 2.3 (m, 4H). **^{13}C NMR** (101 MHz, $CDCl_3$) δ 176.3, 155.6, 152.4, 152.4, 146.7, 145.7, 139.1, 134.7, 128.9, 128.7, 128.1, 127.5, 126.8, 123.9, 121.2, 120.9, 120.7, 120.6, 119.5, 77.5, 77.2, 76.8, 59.0, 38.8, 38.7, 32.0, 31.6. **HRMS** (APCI): **M+H$^+$** Calcd for $C_{24}H_{18}BrO_3^+$: 433.0398 Found: 433.0375.

Synthesis of **6**.

2,6-Dibromo-4-fluorobenzonitrile (**5**, 1.39 g, 5 mmol), carbazole (1.67 g, 10 mmol), bis(dibenzylideneacetone)palladium(0) [Pd(dba)$_2$] (58.9 mg, 0.1 mmol), 2-dicyclohexylphosphino-2',6'-dimethoxybiphenyl (SPhos) (83 mg, 0.2 mmol), and potassium phosphate (K_3PO_4, 3.2 g, 15 mmol) were dissolved in toluene (50 mL). The reaction mixture was stirred under a nitrogen atmosphere and refluxed for 24 h. After completion of the reaction, the mixture was cooled to room temperature and extracted with DCM three times. The combined organic layers were dried over anhydrous sodium sulfate, filtered, and concentrated under reduced pressure to afford the crude product. The crude residue was purified by column chromatography using PE/DCM (3:1, v/v) as the eluent to give the compound **6** as a white solid.

For **6** (1.63g, 77%): **^{1}H NMR** (300 MHz, $CDCl_3$) δ 8.2 (d, J = 7.7 Hz, 4H), 7.6 – 7.5 (m, 6H), 7.4 – 7.4 (m, 8H). **^{13}C NMR** (101 MHz, $CDCl_3$) δ 140.4, 127.2, 126.7, 124.5, 121.7, 121.0, 116.8, 116.6, 109.8. **HRMS** (APCI): **M+H$^+$** Calcd for $C_{31}H_{19}FN_3^+$: 452.1557 Found: 452.1545.

Synthesis of **(*S*)-DCz-PTZ/(*R*)-DCz-PTZ**.

A mixture of **(*S*)/(*R*)-4** (2.17 g, 5 mmol) and K_2CO_3, (4.15 g, 30 mmol) was dissolved in anhydrous *N,N*-dimethylformamide (DMF, 30 mL). Compound **6** (2.13 g, 5 mmol) was then added under stirring. The reaction mixture was heated to 140 °C and stirred for 24 h. After completion of the reaction, the mixture was cooled to room temperature and quenched with saturated NH_4Cl aqueous solution (80 mL). The resulting mixture was extracted with EA three times. The combined organic layers were dried over anhydrous sodium sulfate, filtered, and concentrated under reduced pressure to afford the crude product. The crude residue was purified by column chromatography to give compound **(*S*)-7/(*R*)-7** as the desired intermediate.

Subsequently, compound **(*S*)-7/(*R*)-7** (3.46 g, 4 mmol), phenothiazine (1.21 g, 5 mmol), palladium acetate [Pd(OAc)$_2$] (68 mg, 0.3 mmol), tri-*tert*-butylphosphonium tetrafluoroborate (262 mg, 0.9 mmol), and K_2CO_3 (2.48 g, 18 mmol) were added to

toluene (30 mL). Under a nitrogen atmosphere, the reaction mixture was heated to 120 °C and stirred for 24 h. After completion of the reaction, the mixture was cooled to room temperature and extracted with DCM three times. The combined organic layers were dried over anhydrous sodium sulfate, filtered, and concentrated under reduced pressure to afford the crude product. The crude residue was purified by column chromatography using petroleum ether/ethyl acetate (3:1, v/v), **(*S*)-DCz-PTZ/(*R*)-DCz-PTZ** can be afforded as yellow solid.

After the two-step reaction sequence, the target enantiomers were obtained as yellow solids: **(*S*)-DCz-PTZ** (3.98 g, 81%) and **(*R*)-DCz-PTZ** (3.95 g, 81%). **^{1}H NMR** (500 MHz, $CDCl_3$) δ 8.1 (dd, *J* = 13.2, 7.7 Hz, 4H), 8.0 (d, *J* = 8.0 Hz, 1H), 7.6 (t, *J* = 7.7 Hz, 2H), 7.5 (t, *J* = 7.7 Hz, 2H), 7.4 – 7.3 (m, 8H), 7.3 (d, *J* = 8.0 Hz, 4H), 7.2 (d, *J* = 8.0 Hz, 2H), 7.2 (d, *J* = 8.1 Hz, 2H), 7.1 (d, *J* = 8.0 Hz, 1H), 7.0 (dd, *J* = 7.7, 4.4 Hz, 2H), 6.8 (s, 2H), 6.7 (d, *J* = 7.7 Hz, 2H), 6.2 (dd, *J* = 9.0, 2.3 Hz, 1H), 5.6 (d, *J* = 2.2 Hz, 1H), 3.2 – 3.0 (m, 4H), 2.6 (dt, *J* = 12.8, 9.3 Hz, 1H), 2.4 (d, *J* = 7.2 Hz, 1H), 2.3 (dt, *J* = 12.6, 6.7 Hz, 2H). **^{13}C NMR** (126 MHz, $CDCl_3$) δ 143.5, 140.2, 139.8, 134.6, 129.0, 128.6, 127.9, 127.1, 126.7, 126.4, 126.1, 124.2, 123.9, 122.3, 121.4, 121.3, 120.7, 120.4, 120.3, 118.3, 114.4, 110.7, 109.9, 109.7, 98.7, 58.8, 39.3, 39.2, 32.3. **HRMS** (APCI): **M+H$^+$** Calcd for $C_{67}H_{43}N_4O_3S^+$: 983.3050 Found: 983.3058.

3. Thermal Analysis

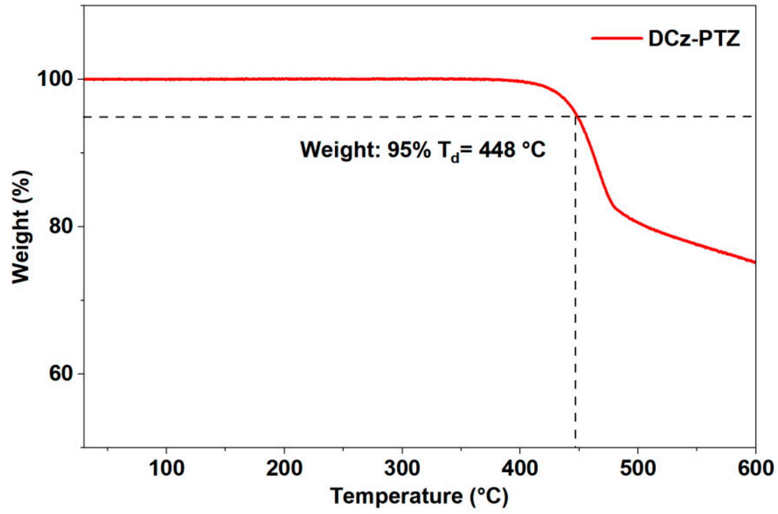

Figure S1. The thermogravimetric analysis of **DCz-PTZ.**

4. Photophysical Properties

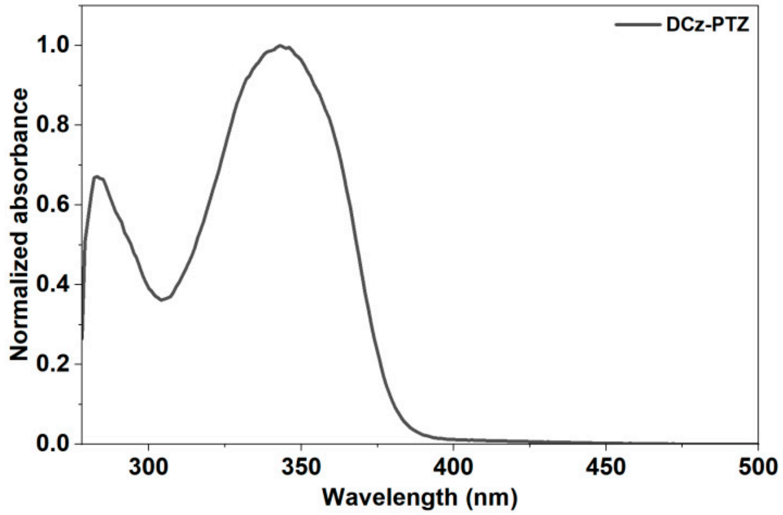

Figure S2. UV-vis absorption spectrum of **DCz-PTZ** in toluene solution ($c=1.0\times10^{-5}$ M, L=1 cm). The maximum molar absorption coefficient was estimated to be 5.91×10^{4} $M^{-1}cm^{-1}$ according to the Beer-Lambert law.

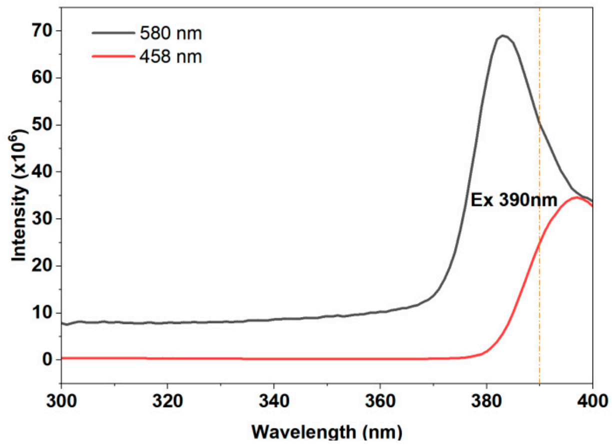

Figure S3. Excitation spectra of **DCz-PTZ** at different emission wavelengths in dilute toluene solution at 298 K.

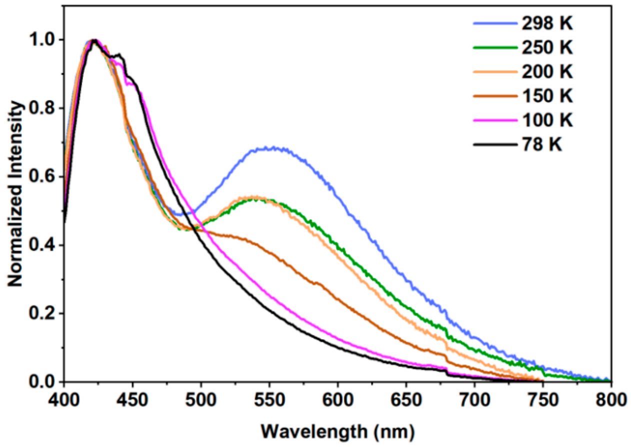

Figure S4. Temperature dependent steady-state fluorescence spectra of **DCz-PTZ** in doped films.

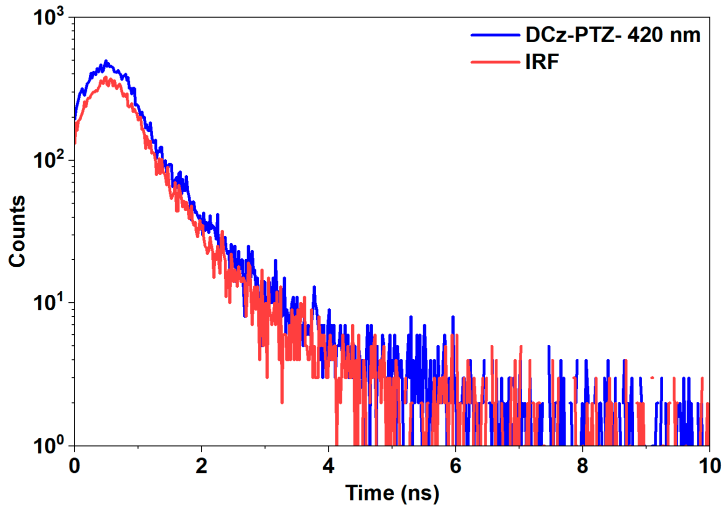

Figure S5. High-resolution transient PL decay profile of DCz-PTZ monitored at 420 nm with the corresponding instrument response function (IRF).

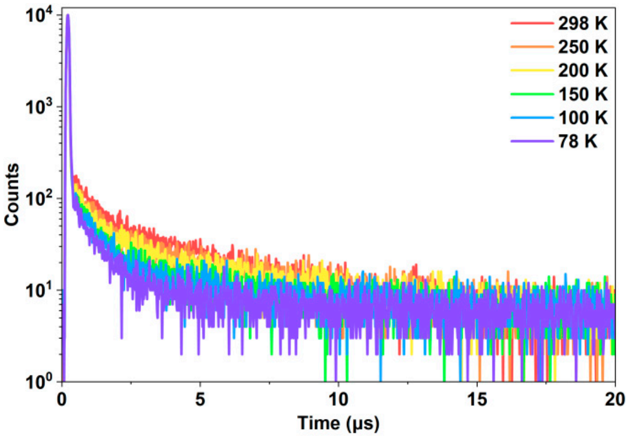

Figure S6. Temperature dependent transient fluorescence spectra of **DCz-PTZ** in doped films.

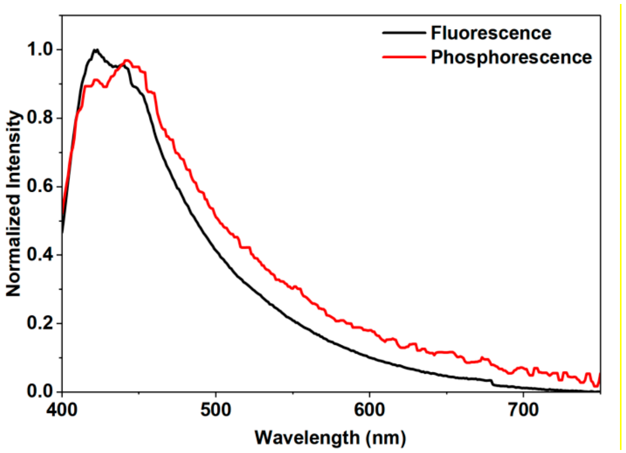

Figure S7. Fluorescence and phosphorescence spectra of **DCz-PTZ** in doped films at 77 K.

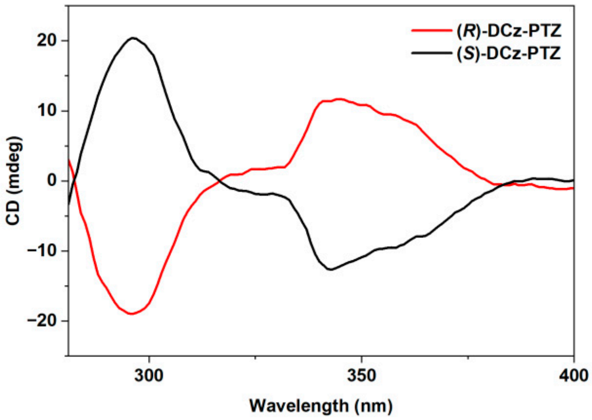

Figure S8. CD spectrum of (*R*/*S*)-**DCz-PTZ** in dilute toluene solution at 298 K.

Table S1. Chiroptical properties of representative chiral TADF emitters.

Compound	Luminescence dissymmetry factor	Ref.
(*R/S*)-DO-PTZ	3.0×10^{-3}	*Angew. Chem. Int. Ed.* **2024**, *63*: e202409020
(*R/S*)-o-NA,	$<0.5 \times 10^{-3}$	*Angew. Chem. Int. Ed.* **2025**, *64*: e202420474
(*R/S*)-**SDMAC**	1.39×10^{-3}	*Angew. Chem. Int. Ed.* **2022**, *61*: e202206861
(*R*)/(*S*)-BIT	5.0×10^{-3}	*Advanced Optical Materials* **2024**, *12*: 2302486

5. Theoretical Calculations

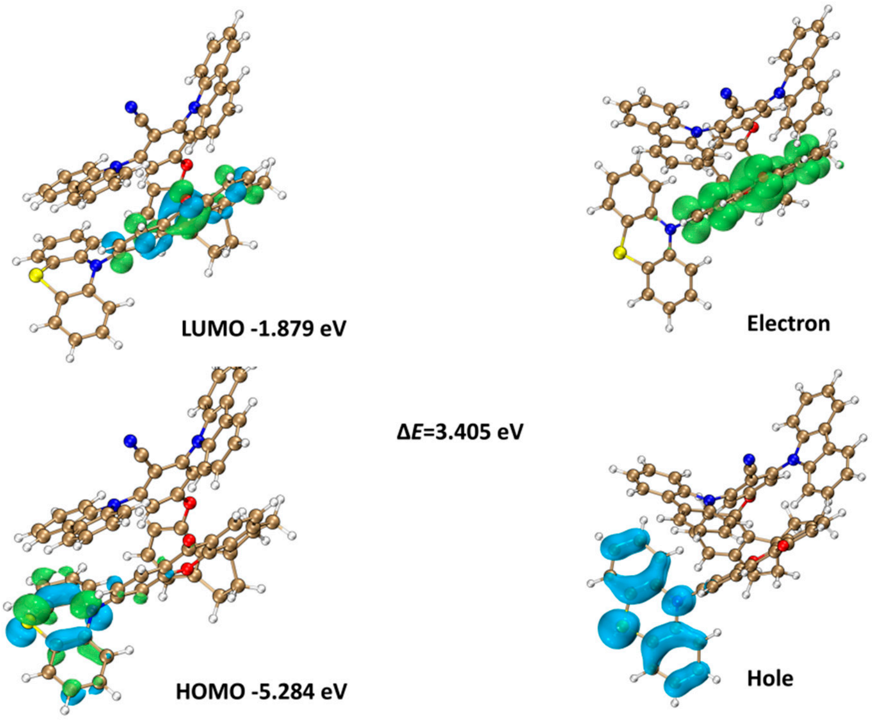

Figure S9. Corresponding HOMO, LUMO, electron and hole distributions and calculated energy levels of DCz-PTZ based on optimized structure (DFT with B3LYP functional and the 6-31G(d) basis set)

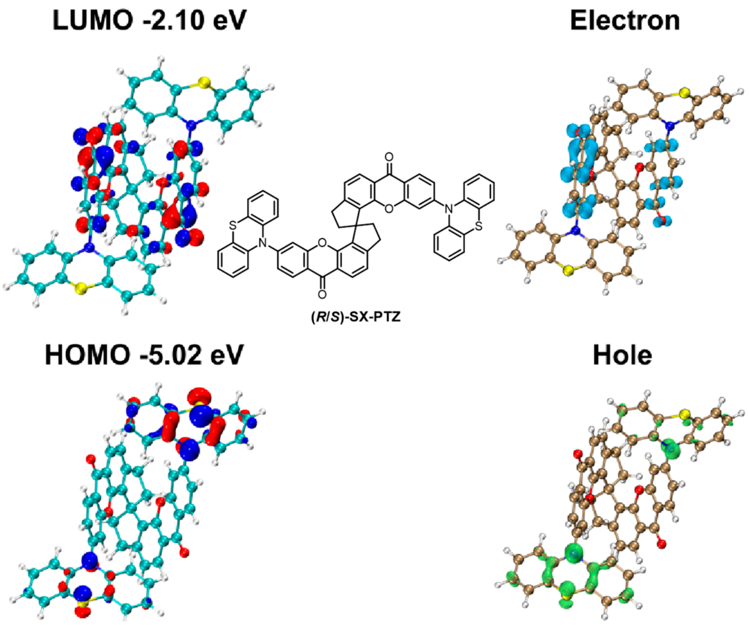

Figure S10. Calculated HOMO/LUMO distributions and NTO hole/electron distributions of the reference molecule **SX-PTZ** without effective π-interrupted/spiro decoupling. The spatial separation between the hole and electron distributions indicates a pronounced CT character. Combined with the insufficient blue-emission component observed in its PL spectrum, this result suggests that the absence of effective π interruption leads to CT-dominated emission and suppresses the high-energy locally excited component.

Table S2. Comparison of excited-state characteristics and PL behavior between DCz-PTZ and the reference molecule **SX-PTZ**.

Molecule	Structural feature	Calculated excited-state feature	PL behavior in solvent	Implication
SX-PTZ	without effective π interruption	CT-dominated; separated hole/electron	insufficient blue emission	high-energy local component suppressed
DCz-PTZ	spiro/π-interrupted framework	LE/CT coexistence; DCz–CN-related high-energy component	dual emission / white emission	LE-involved and CT channels balanced

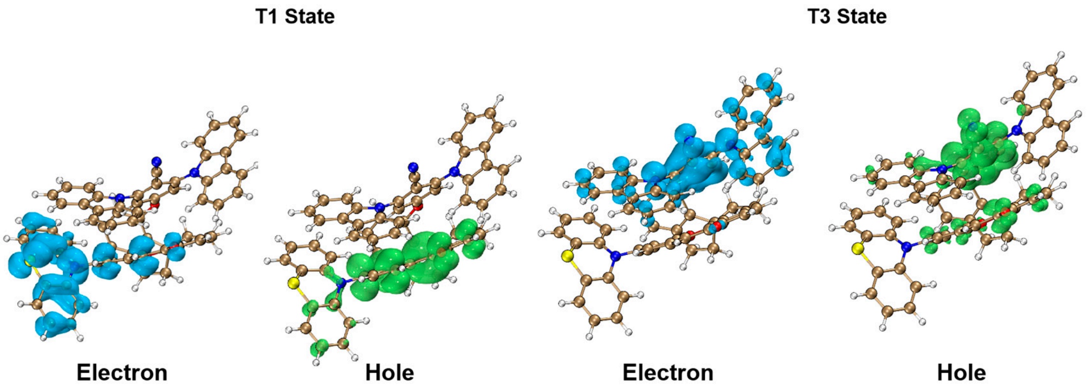

Figure S11. Natural transition orbital distributions of the T1 and T3 excited states of DCz-PTZ calculated by TD-DFT.

6. Device Characterization

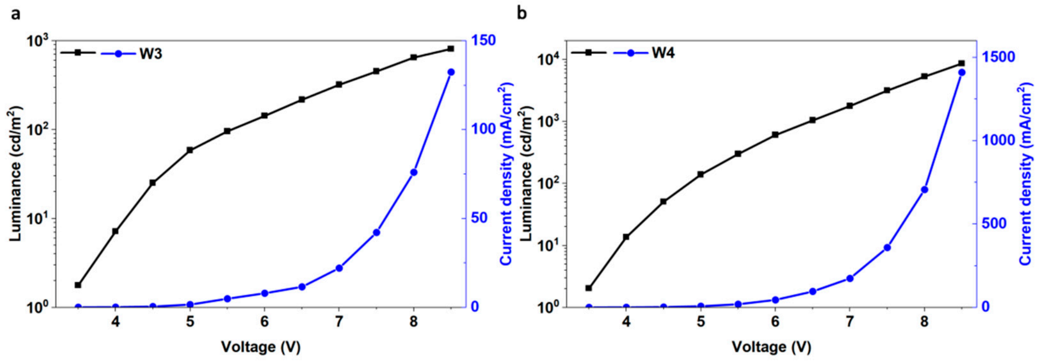

Figure S12. a) Current density-voltage-Luminance (*J*-*V*-*L*) characteristics of devices W3 b) *J*-*V*-*L* characteristics of devices W4.

7. NMR Spectra of New Products

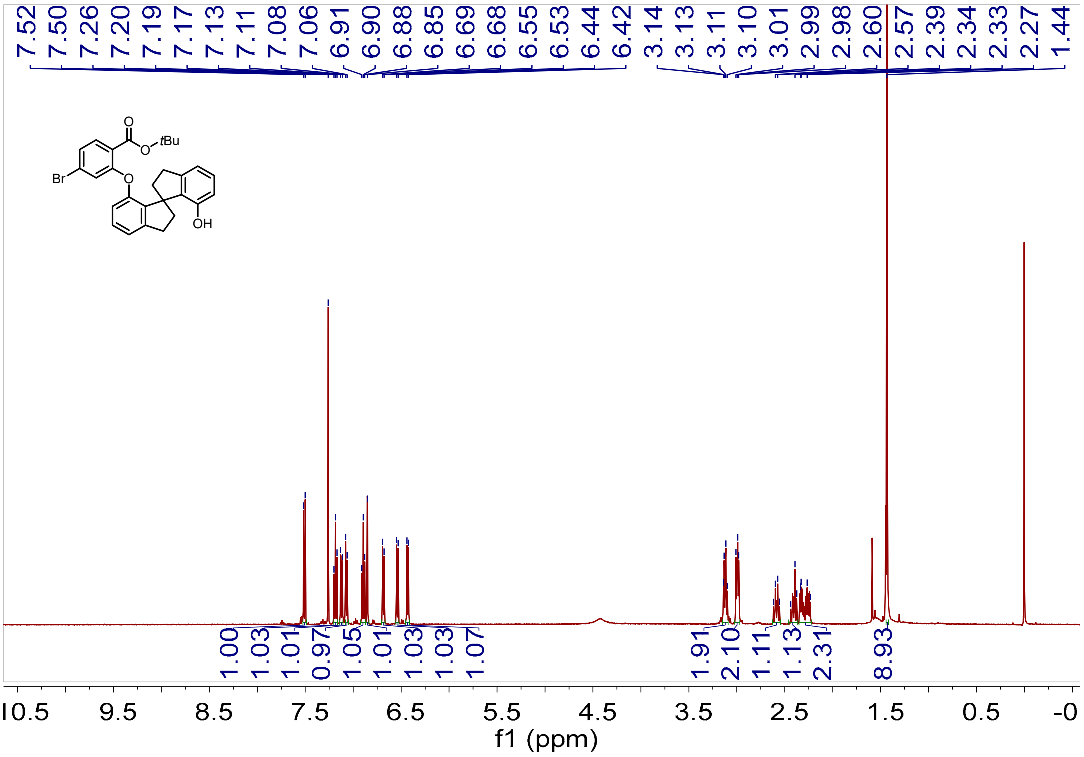

Figure S13. ^{1}H NMR spectrum of **(*S*)-3/(*R*)-3** (500 MHz, $CDCl_3$).

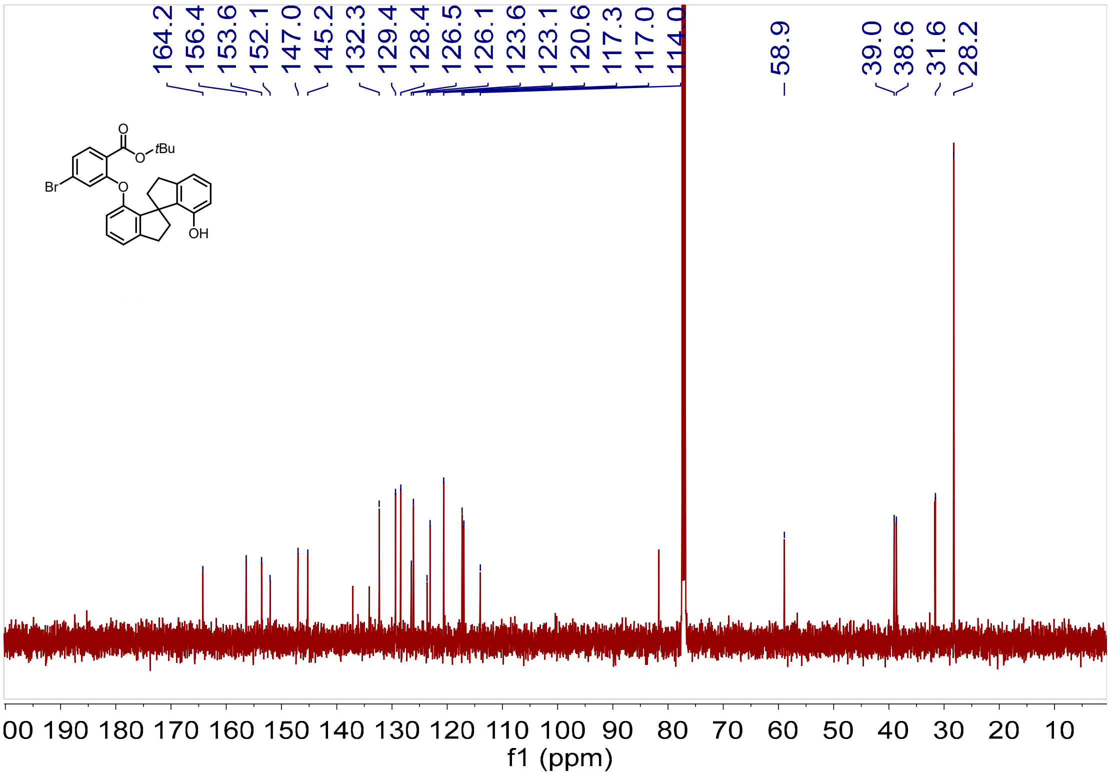

Figure S14. ^{13}C NMR spectrum of **(*S*)-3/(*R*)-3** (126 MHz, $CDCl_3$).

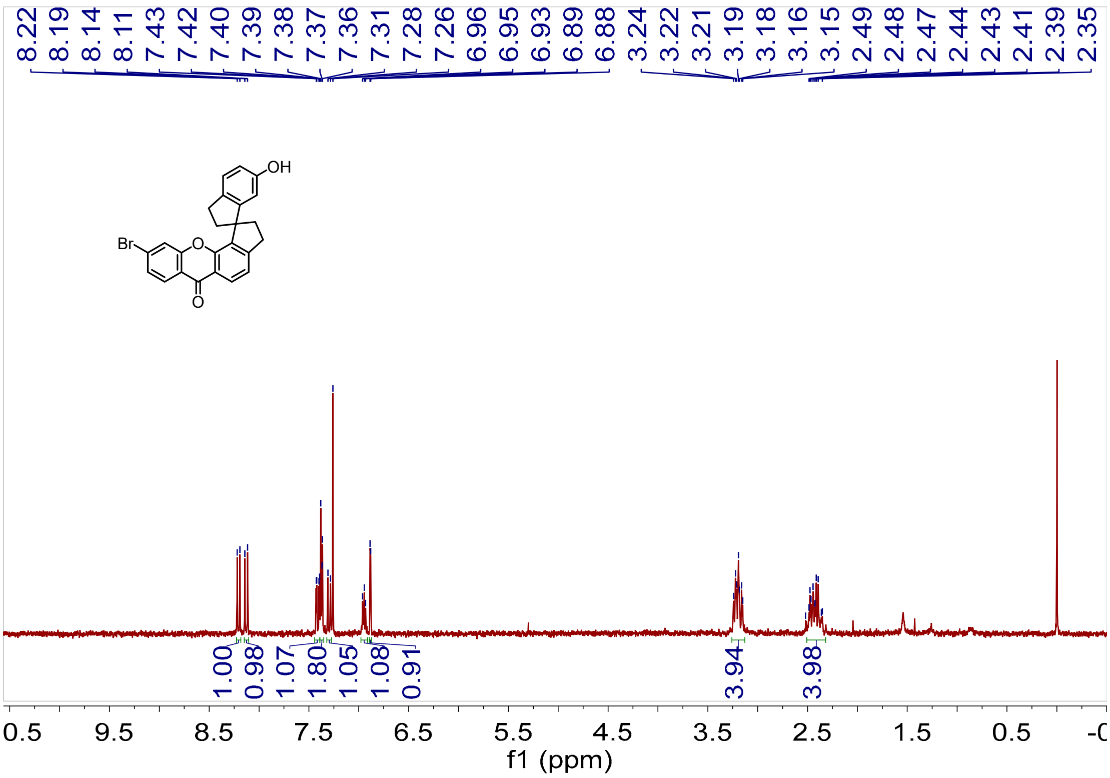

Figure S15. ^{1}**H NMR** spectrum of **(*S*)-4/(*R*)-4** (300 MHz, $CDCl_3$).

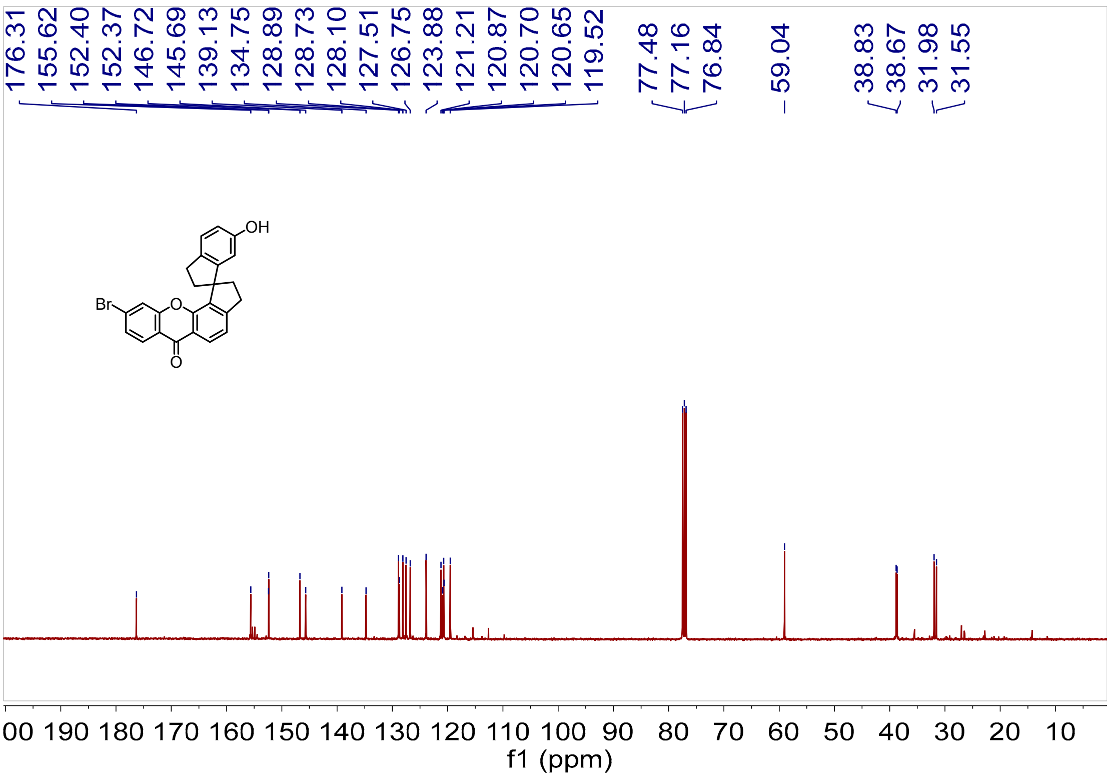

Figure S16. ^{13}**C NMR** spectrum of **(*S*)-4/(*R*)-4** (100 MHz, $CDCl_3$).

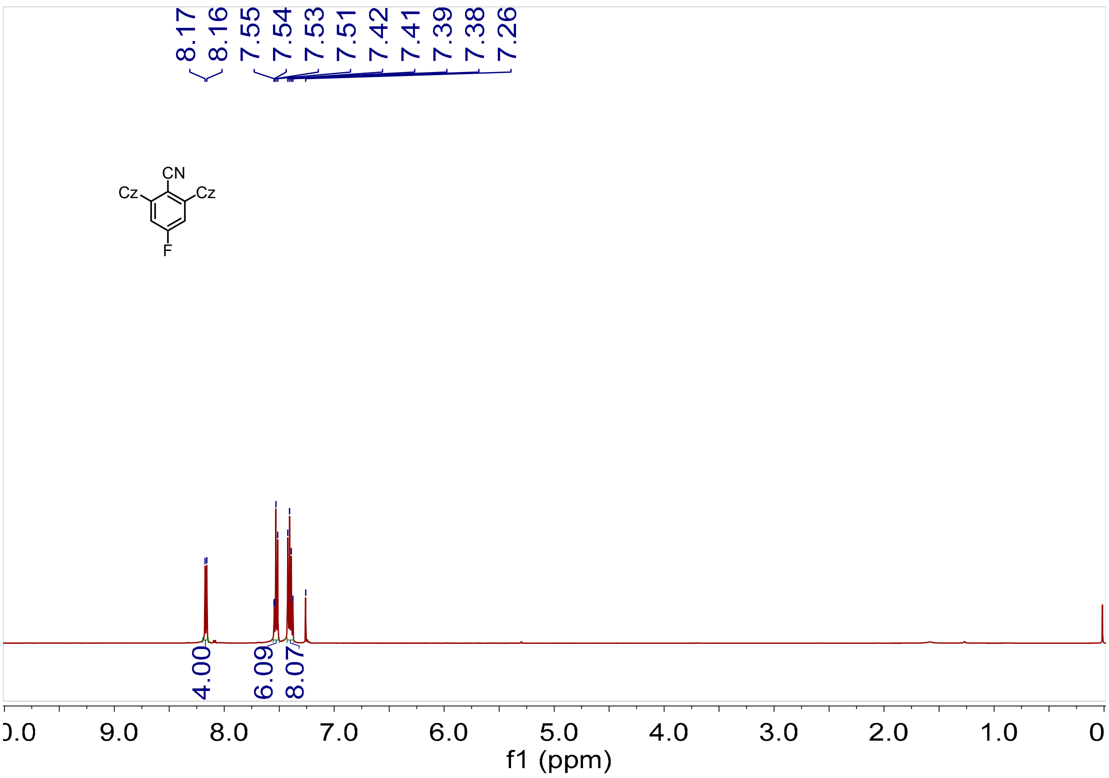

Figure S17. ^{1}**H NMR** spectrum of compound **6** (300 MHz, $CDCl_3$).

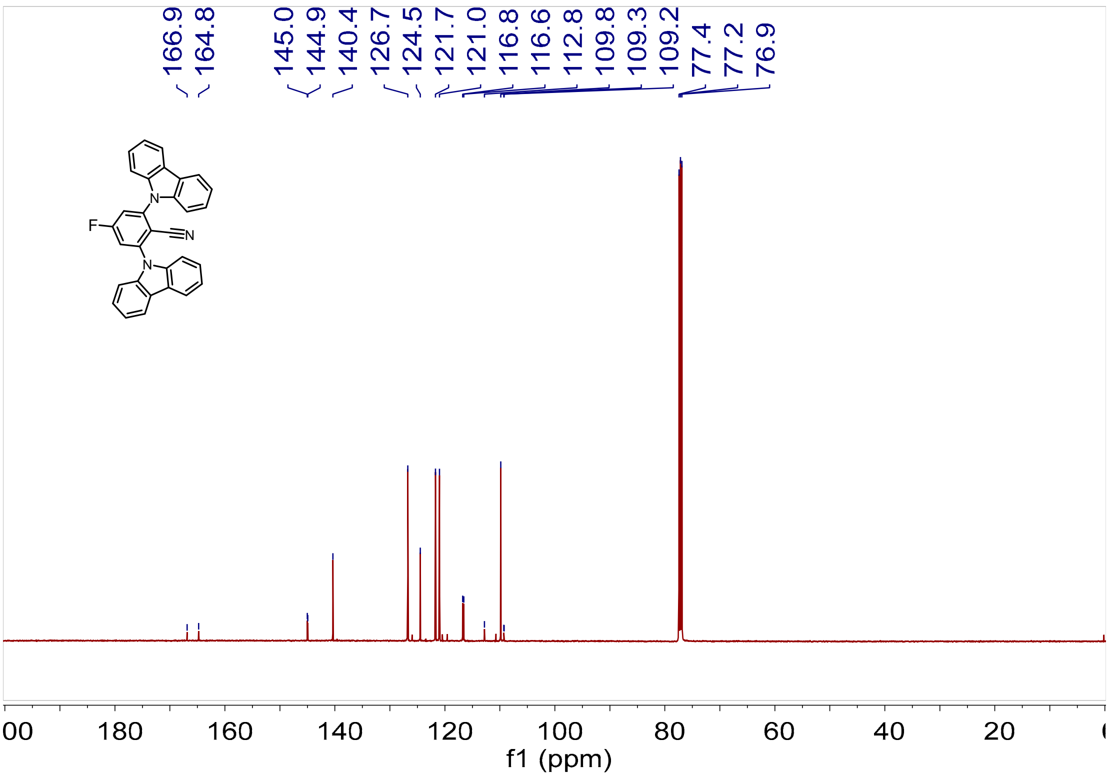

Figure S18. ^{13}**C NMR** spectrum of compound **6** (100 MHz, $CDCl_3$).

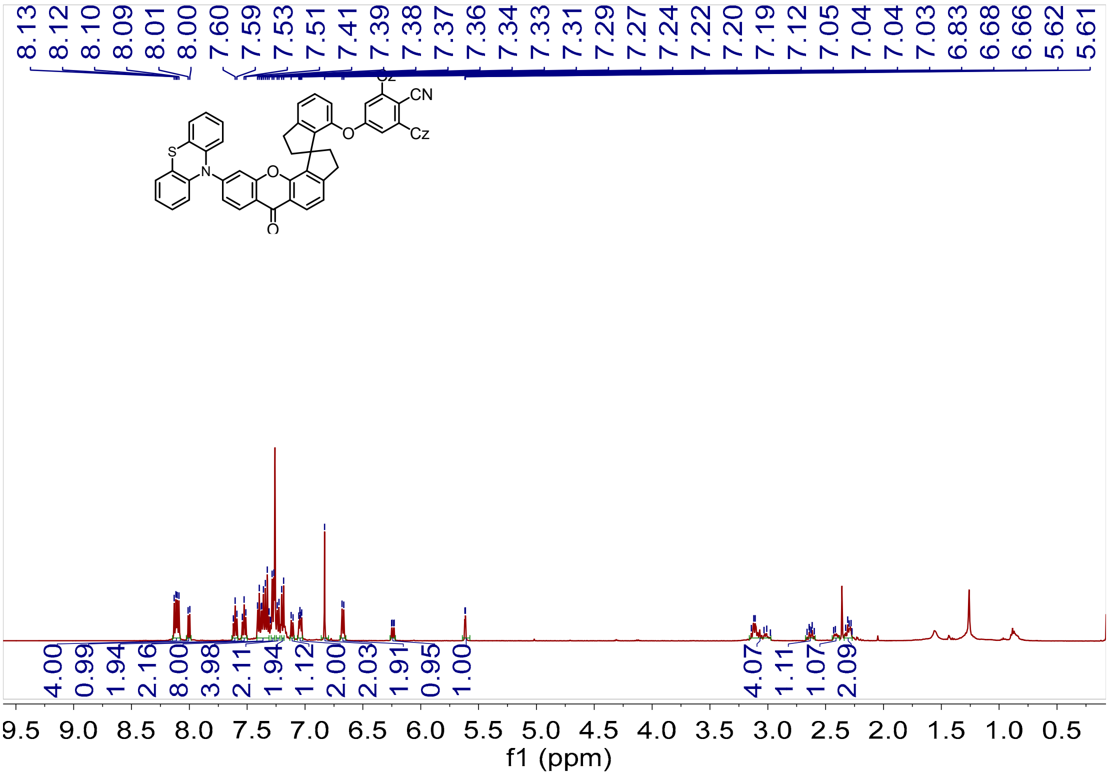

Figure S19. ^{1}**H NMR** spectrum of compound **(*S*)-DCz-PTZ/(*R*)-DCz-PTZ** (500 MHz, $CDCl_3$).

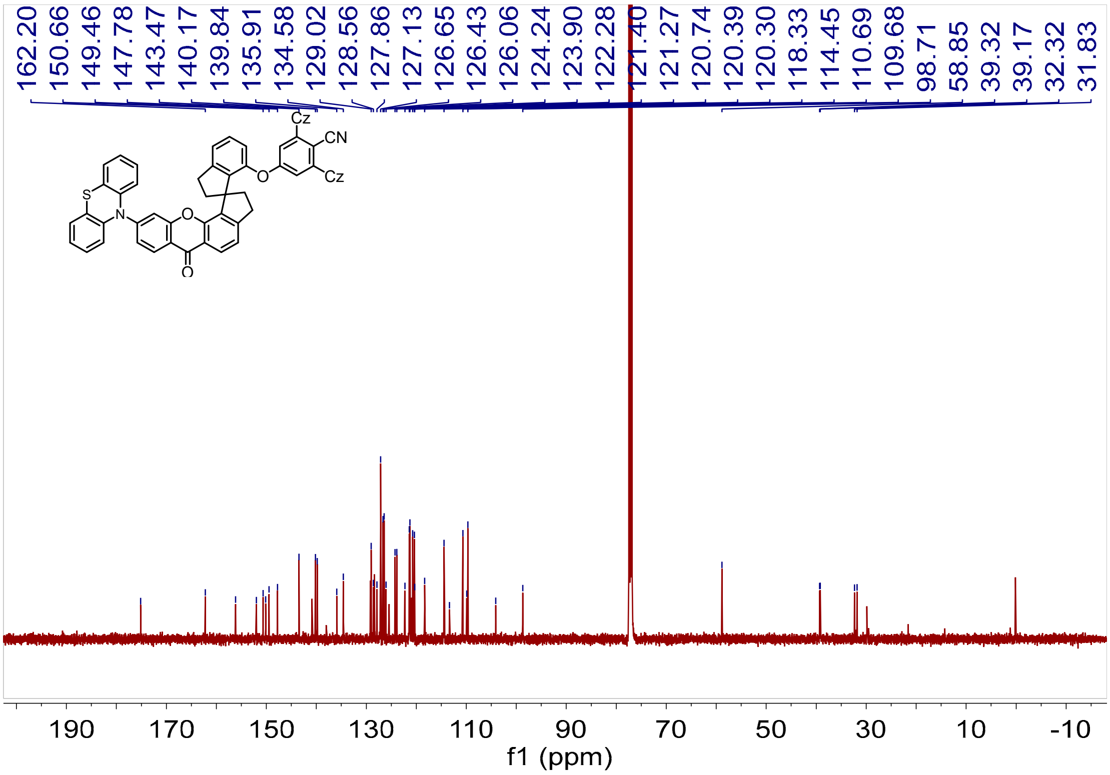

Figure S20. ^{13}**C NMR** spectrum of compound **(*S*)-DCz-PTZ/(*R*)-DCz-PTZ** (126 MHz, $CDCl_3$).

8. References

39 Gaussian 09, Revision D.01, M. J. Frisch, G. W. Trucks, H. B. Schlegel, G. E. Scuseria, M. A. Robb, J. R. Cheeseman, G. Scalmani, V. Barone, B. Mennucci, G. A. Petersson, H. Nakatsuji, M. Caricato, X. Li, H. P. Hratchian, A. F. Izmaylov, J. Bloino, G. Zheng, J. L. Sonnenberg, M. Hada, M. Ehara, K. Toyota, R. Fukuda, J. Hasegawa, M. Ishida, T. Nakajima, Y. Honda, O. Kitao, H. Nakai, T. Vreven, J. A. Montgomery, Jr., J. E. Peralta, F. Ogliaro, M. Bearpark, J. J. Heyd, E. Brothers, K. N. Kudin, V. N. Staroverov, T. Keith, R. Kobayashi, J. Normand, K. Raghavachari, A. Rendell, J. C. Burant, S. S. Iyengar, J. Tomasi, M. Cossi, N. Rega, J. M. Millam, M. Klene, J. E. Knox, J. B. Cross, V. Bakken, C. Adamo, J. Jaramillo, R. Gomperts, R. E. Stratmann, O. Yazyev, A. J. Austin, R. Cammi, C. Pomelli, J. W. Ochterski, R. L. Martin, K. Morokuma, V. G. Zakrzewski, G. A. Voth, P. Salvador, J. J. Dannenberg, S. Dapprich, A. D. Daniels, O. Farkas, J. B. Foresman, J. V. Ortiz, J. Cioslowski, and D. J. Fox, Gaussian, Inc., Wallingford CT, **2013**.

40 S. Grimme, J. Antony, S. Ehrlich, H. Krieg, *J. Chem. Phys*. **2010**, *132*, 154104.

41 F. Weigend, R. Ahlrichs, Phys. Chem. *Chem. Phys*. **2005**, *7*, 3297.

42 S. Grimme, S. Ehrlich, L. Goerigk, *J. Comput. Chem*. **2011**, *32*, 1456-1465.

43 P. J. Stephens, F. J. Devlin, C. F. Chabalowski, M. J. Frisch, *J. Phys. Chem*. **1994**, *98*, 11623-11627.

44 J. Liu, T. He, Z.-L. Gong, N. Liang, Y. Feng, G. Long, Y.-W. Zhong, C.-J. Yao, *Adv. Optical Mater.* **2024**, *12*, 2302486.
